# Pseudotime-conditioned diffusion models for imputing time-series single-cell data

**DOI:** 10.1093/bioadv/vbag248

**Published:** 2026-08-24

**Authors:** Graham Bishop, Min-Ho Ku, Daniel Lee, Haijun Gong, Tong Si

**Affiliations:** Department of Mathematics and Statistics, Saint Louis University, Saint Louis, MO 63103, United States; School for Professional Studies, Saint Louis University, Saint Louis, MO 63103, United States; Department of Mathematics and Statistics, Saint Louis University, Saint Louis, MO 63103, United States; Department of Mathematics and Statistics, Saint Louis University, Saint Louis, MO 63103, United States; Department of Family and Community Medicine, Saint Louis University, Saint Louis, MO 63103, United States

## Abstract

**Motivation:**

Probabilistic diffusion-based imputation methods have demonstrated superior performance over many state-of-the-art approaches for imputing single-cell data. However, existing methods typically rely on isotropic Gaussian noise and canonical observation time, limiting their ability to capture structured temporal dependencies and continuous cellular dynamics. In time-series single-cell RNA-seq (scRNA-seq) data, these limitations are exacerbated by the small number of experimentally measured time points and the substantial heterogeneity among cells collected at the same time point, causing observed time labels to poorly reflect true biological progression.

**Results:**

To address these challenges, we propose the first pseudotime-conditioned diffusion-based imputation framework for time-series scRNA-seq data, consisting of two trajectory-aware models: pseudotime-conditioned conditional score-based diffusion imputation (P-CSDI) and pseudotime-conditioned hybrid-noise CSDI (PH-CSDI). P-CSDI incorporates inferred pseudotime as a conditioning signal to guide the denoising process with biologically meaningful temporal structure, while PH-CSDI further introduces hybrid structured noise to improve frequency-aware temporal modeling. By leveraging pseudotime as a continuous representation of cellular progression, both models enable trajectory-aware and temporally coherent reconstruction of missing gene expression values. P-CSDI and PH-CSDI consistently reduce mean absolute error and root mean squared error by approximately 40%–60% compared with state-of-the-art imputation methods across two scRNA-seq datasets under diverse missingness settings. These findings highlight the importance of incorporating biologically informed temporal structure into diffusion-based generative models for accurate imputation of dynamic single-cell transcriptomic data.

**Availability and implementation:**

The computer code and data for the proposed method are available on GitHub: https://github.com/gbishop345/pseudotime-CSDI.

## 1 Introduction

Single-cell RNA sequencing (scRNA-seq) provides high-resolution measurements of gene expression across different tissues at the cellular level over multiple time points (Potter 2018, Karlsson *et al*. 2021). Analysis of time-series scRNA-seq data offers valuable insights into heterogeneous cell populations and the dynamic cellular processes underlying complex biological systems and diseases (Trapnell *et al*. 2014, Maizels 2024). However, a major challenge in time-series scRNA-seq analysis is the pervasive presence of missing values caused by technological limitations such as dropout events (Zhang and Zhang 2021). The combination of high missingness, limited temporal resolution and the inherently high dimensionality of scRNA-seq data substantially compromises the reliability of downstream analyses (Kharchenko *et al*. 2014, Lähnemann *et al*. 2020).

To address missing value imputation in high-dimensional single-cell data, numerous methods have been developed, including traditional imputation approaches [e.g. Multivariate Imputation by Chained Equations (MICE) and K-Nearest Neighbors (KNN)] (Batista and Monard 2002, Buuren and Groothuis-Oudshoorn 2011), graph diffusion-based methods (e.g. Markov Affinity-based Graph Imputation of Cells (MAGIC)) (Van Dijk *et al*. 2018), and generative model-based techniques (Yoon *et al*. 2018, Liu *et al*. 2023, Si *et al*. 2023, Zhang *et al*. 2024, Bishop *et al*. 2025). Traditional methods often rely on restrictive assumptions, limiting their ability to capture the complex nonlinear gene–gene dependencies present in single-cell transcriptomic data (Platias and Petasis 2020). Recent advances in deep generative modeling, including variational autoencoders (VAEs) (Kingma 2013), generative adversarial networks (GANs) (Goodfellow *et al*. 2014), and probabilistic diffusion models (Ho *et al*. 2020), have enabled more flexible modeling of complex data distributions. Representative VAE- and GAN-based imputation methods include GP-VAE (Fortuin *et al*. 2020), VIGAN (Shang *et al*. 2017), T-LSTM-VAE (Xu *et al*. 2026), sc-fGAIN (Si *et al*. 2023), SGAIN (Neves *et al*. 2021), along with time-series extensions that incorporate recurrent or attention mechanisms (Luo *et al*. 2018, Oh *et al*. 2021, Liu *et al*. 2025). However, GAN-based approaches remain susceptible to training instability and sensitivity to the choice of adversarial divergence (Si *et al*. 2023).

Diffusion-based imputation methods (Tashiro *et al*. 2021, Chen *et al*. 2023, Zhou *et al*. 2024), such as the conditional score-based diffusion model for imputation (CSDI) (Tashiro *et al*. 2021), Frequency-Shaped Diffusion (FSDI) (Shen *et al*. 2026), and Multivariate Time Series Consistent Imputation (MTSCI) (Zhou *et al*. 2024), have demonstrated superior performance over GAN-based approaches due to their stable training dynamics and strong ability to model complex high-dimensional distributions through iterative noising and denoising. Nevertheless, most existing diffusion-based imputation methods rely on isotropic Gaussian noise, which perturbs all signal components uniformly and ignores structured temporal dependencies in sequential data. To address this limitation, our recent work (Bishop *et al*. 2025) introduced a time-varying blue noise-based conditional score-based diffusion model (tBN-CSDI) for time-series imputation. By incorporating structured blue noise into the diffusion process, tBN-CSDI better preserves temporal correlations and captures fine-grained high-frequency variations, resulting in superior performance compared to conventional Gaussian-noise-based diffusion models. These findings highlight that temporal structure plays a critical role in accurately recovering missing values in time-series data.

However, effectively learning temporal structure remains challenging in time-series scRNA-seq data because the number of experimentally measured time points is often limited, and cells collected at the same time point may correspond to heterogeneous developmental states (Trapnell *et al*. 2014, Street *et al*. 2018). As a result, canonical observation time may poorly reflect true biological progression, creating a mismatch between observed time and underlying cellular dynamics. Consequently, generative models that rely solely on canonical time or static representations may fail to accurately recover dynamic gene expression patterns and temporal regulatory relationships. Pseudotime analysis (Saelens *et al*. 2019) provides an effective framework for characterizing latent temporal structure in time-series single-cell data by ordering cells along continuous developmental trajectories based on gene expression similarity, thereby offering a high-resolution representation of cellular progression beyond discrete experimental time labels. Numerous trajectory inference methods have been developed for this purpose, including Monocle (Trapnell *et al*. 2014), Slingshot (Street *et al*. 2018), PHATE (Moon *et al*. 2019), and diffusion-based approaches such as diffusion pseudotime (DPT) (Haghverdi *et al*. 2016). Prior studies (Zhang *et al*. 2025) have shown that incorporating pseudotime information can improve downstream analyses such as dynamic gene regulatory network inference by providing biologically meaningful temporal ordering of cells.

Despite these advances, pseudotime has rarely been integrated into deep generative frameworks for missing value imputation, and existing diffusion-based imputation methods remain disconnected from trajectory inference. To address this gap, we propose incorporating pseudotime directly into the diffusion process as a conditioning variable, enabling the model to leverage inferred cellular trajectories for temporally coherent and biologically informed imputation. Specifically, we develop two pseudotime-conditioned score-based diffusion models for imputation, pseudotime-conditioned conditional score-based diffusion imputation (P-CSDI) and pseudotime-conditioned hybrid-noise CSDI (PH-CSDI), which integrate pseudotime as a conditioning variable in the denoising/imputation process, with PH-CSDI further incorporating hybrid noise mechanisms to enhance frequency-aware modeling. By leveraging pseudotime as a continuous representation of cellular progression beyond discrete experimental time labels, the proposed framework enables trajectory-aware and temporally coherent reconstruction of missing gene expression values, improving biologically meaningful imputation and the modeling of dynamic cellular processes. To our knowledge, this is the first study to integrate pseudotime information into the diffusion-based imputation frameworks for time-series scRNA-seq data. We evaluate the proposed frameworks on two time-series scRNA-seq datasets and demonstrate that the pseudotime-conditioned diffusion models consistently outperform several state-of-the-art imputation methods across diverse experimental settings, highlighting the importance of incorporating biologically informed temporal structure into generative models for accurate imputation of dynamic single-cell transcriptomic data.

## 2 Methods

Let $X\in R^{K\times L}$ denote a multivariate time series with *K* features measured over *L* time points, and let M be the corresponding binary mask indicating observed (1) and missing 0 entries. Let $s\in R^{L}$ represent the time intervals between consecutive observations. The imputation task aims to model the conditional distribution of the missing values $X^{\text{mis}}$ given the observed data $X^{\text{obs}}$, the mask, and temporal information: $p(X^{\text{mis}}|X^{\text{obs}},M,s)$. Given the superior performance of probabilistic diffusion-based methods in modeling complex high-dimensional distributions, we focus on diffusion-based generative frameworks for time-series imputation.

Existing diffusion-based imputation methods are formulated using canonical observation time, which often fails to capture biologically meaningful temporal structure. However, in time-series single-cell data, cells measured at the same time point may correspond to different biological states, making canonical time an inadequate representation of underlying cellular dynamics. To address this limitation, we propose pseudotime-guided conditional diffusion frameworks for imputation, which leverages inferred cellular trajectories to capture biologically relevant progression. Building upon denoising diffusion probabilistic models (DDPMs) (Ho *et al*. 2020), the CSDI (Tashiro *et al*. 2021), and our prior tBN-CSDI framework (Bishop *et al*. 2025), we further incorporate both time-varying hybrid noise and pseudotime information to better capture temporal dependencies and improve biologically coherent imputation.

Next, we will introduce the following key components of our frameworks: (i) the pseudotime-conditioned denoising diffusion model, (ii) the pseudotime-conditioned diffusion model for imputation (P-CSDI), (iii) the PH-CSDI, and (iv) pseudotime estimation procedure.

### 2.1 Pseudotime-conditioned denoising diffusion model

Given single-cell data, each sample $x_{0}$ is assigned a pseudotime coordinate τ∈R, inferred from different trajectory reconstruction methods such as Monocle (Trapnell *et al*. 2014), Slingshot (Street *et al*. 2018), or DPT (Haghverdi *et al*. 2016). The pseudotime value τ represents the cell’s position along a latent biological trajectory. Our goal is to model the conditional data distribution $q(x_{0}|\tau )$, where pseudotime serves as a continuous conditioning variable, rather than the unconditional distribution $q(x_{0})$ considered in traditional diffusion models (Ho *et al*. 2020).

The proposed framework consists of a forward diffusion process and a reverse denoising process. The forward process progressively perturbs the data with Gaussian noise, while the reverse process learns to reconstruct the original data conditioned on pseudotime τ. To preserve the tractability of the standard diffusion formulation, pseudotime is incorporated only in the reverse denoising process and does not modify the forward noising dynamics. Accordingly, the forward diffusion process follows the standard formulation of DDPMs, in which Gaussian noise is progressively added to the original data through the following Markov chain:


$$\begin{array}{cc} q(x_{1:T}|x_{0}) & =\prod_{t=1}^{T}q(x_{t}|x_{t-1}),\\ q(x_{t}|x_{t-1}) & =N\left(x_{t};\sqrt{\alpha_{t}}x_{t-1},(1-\alpha_{t})I\right), \end{array}$$

where $\alpha_{t}\in (0,1)$ controls the noise level at diffusion step *t*.

By recursively applying this process, the marginal distribution of $x_{t}$ conditioned on the original data $x_{0}$ can be expressed in closed form as


$$q(x_{t}|x_{0})=N\left(x_{t};\sqrt{{\bar{\alpha }}_{t}}x_{0},(1-{\bar{\alpha }}_{t})I\right),$$

where ${\bar{\alpha }}_{t}=\prod_{s=1}^{t}\alpha_{s}$. Equivalently, $x_{t}$ can be sampled directly as


(1) $$x_{t}=\sqrt{{\bar{\alpha }}_{t}}x_{0}+\sqrt{1-{\bar{\alpha }}_{t}}\epsilon ,\;\epsilon ∼N(0,I),$$

thereby enabling efficient sampling without iterating through all intermediate diffusion steps.

Pseudotime is incorporated exclusively in the reverse process to enable trajectory-aware denoising and improve the reconstruction of temporal gene expression dynamics. Accordingly, the reverse diffusion process is formulated as a pseudotime-conditioned Markov chain:


$$p_{\theta }(x_{0:T}|\tau )=p(x_{T})\prod_{t=1}^{T}p_{\theta }(x_{t-1}|x_{t},\tau ).$$

Each transition is modeled as a Gaussian distribution:


$$p_{\theta }(x_{t-1}|x_{t},\tau )=N\left(x_{t-1};\mu_{\theta }(x_{t},t,\tau ),\sigma_{t}^{2}\right),$$

where, the mean function is parameterized as


(2) $$\mu_{\theta }(x_{t},t,\tau )=\frac{1}{\sqrt{\alpha_{t}}}\left(x_{t}-\frac{1-\alpha_{t}}{\sqrt{1-{\bar{\alpha }}_{t}}}\epsilon_{\theta }(x_{t},t|\tau )\right),$$

where $\epsilon_{\theta }(x_{t},t|\tau )$ represents a neural network that predicts the added noise conditioned on both the diffusion step *t* and the pseudotime τ, and $\sigma_{t}^{2}=\frac{\left(1-{\bar{\alpha }}_{t-1})(1-\alpha_{t}\right)}{1-{\bar{\alpha }}_{t}}I$ controls stochasticity in the reverse step.

The pseudotime-conditioned diffusion model is trained by minimizing the following objective function:


(3) $$\underset{\theta }{\min}E_{x_{0},\tau ,\epsilon ,t}\left[{\left\|\epsilon -\epsilon_{\theta }(x_{t},t|\tau )\right\|}_{2}^{2}\right].$$

Compared with standard diffusion models based on canonical observation time, conditioning on pseudotime τ enables the model to learn trajectory-aware denoising functions that vary smoothly along the underlying cellular progression. Consequently, the pseudotime-conditioned diffusion model can be extended for missing value imputation, improving the recovery of biologically coherent temporal dynamics, as described in the next subsection.

### 2.2 P-CSDI: Pseudotime-conditioned diffusion model for imputation

Building upon the pseudotime-conditioned diffusion framework, we extend the CSDI (Tashiro *et al*. 2021) by incorporating pseudotime as an additional conditioning variable, resulting in a pseudotime-conditioned diffusion model for imputation (P-CSDI). Given an input $x_{0}$ with missing entries, we decompose it into observed and missing components, denoted by $x_{0}^{\text{obs}}$ and $x_{0}^{\text{mis}}$, respectively. The objective is to learn the conditional distribution of missing values given the observed entries and pseudotime coordinate τ:


(4) $$p_{\theta }(x_{0}^{\text{mis}}|x_{0}^{\text{obs}},\tau ).$$

Following the vanilla CSDI framework, the forward diffusion process perturbs only the missing components while keeping the observed entries fixed. As in the pseudotime-conditioned diffusion framework described above, pseudotime is not incorporated into the forward noising process. Specifically, we have


(5) $$x_{t}^{\text{obs}}=x_{0}^{\text{obs}},$$

(6) $$x_{t}^{\text{mis}}=\sqrt{{\bar{\alpha }}_{t}}x_{0}^{\text{mis}}+\sqrt{1-{\bar{\alpha }}_{t}}\epsilon .$$

The reverse process is formulated as a pseudotime-conditioned Markov chain, which is expressed as


$$p_{\theta }(x_{0:T}^{\text{mis}}|x_{0}^{\text{obs}},\tau )=p(x_{T}^{\text{mis}})\prod_{t=1}^{T}p_{\theta }(x_{t-1}^{\text{mis}}|x_{t}^{\text{mis}},x_{0}^{\text{obs}},\tau ).$$

Each reverse transition for the missing components is modeled as a Gaussian distribution:


$$p_{\theta }(x_{t-1}^{\text{mis}}|x_{t}^{\text{mis}},x_{0}^{\text{obs}},\tau )=N(x_{t-1}^{\text{mis}};\;\mu_{\theta }(x_{t},t,\tau ),\sigma_{t}^{2}),$$

where the reverse mean is parameterized by a pseudotime-dependent neural network as


$$\mu_{\theta }(x_{t},t,\tau )=\frac{1}{\sqrt{\alpha_{t}}}\left(x_{t}^{\text{mis}}-\frac{1-\alpha_{t}}{\sqrt{1-{\bar{\alpha }}_{t}}}\epsilon_{\theta }(x_{t}^{\text{mis}},t|x_{0}^{\text{obs}},\tau )\right),$$

and $\epsilon_{\theta }(x_{t}^{\text{mis}},t|x_{0}^{\text{obs}},\tau )$ predicts the injected noise for the missing entries conditioned on the diffusion step *t*, observed values, and pseudotime τ.

The pseudotime-conditioned diffusion model for imputation (P-CSDI) is trained by minimizing the following objective:


(7) $$\underset{\theta }{\min}E_{x_{0},\tau ,\epsilon ,t}\left[{\left\|\epsilon -\epsilon_{\theta }(x_{t}^{\text{mis}},t|x_{0}^{\text{obs}},\tau )\right\|}_{2}^{2}\right].$$

Algorithm 1 summarizes the overall training and imputation procedures of the proposed P-CSDI framework for pseudotime-guided trajectory-aware imputation. First, pseudotime is estimated from the input data and used to reorder cells along the inferred developmental trajectory. During training, Gaussian noise is added only to missing entries, and the denoising network is trained to predict the injected noise conditioned on the observed values, diffusion step, and pseudotime. During inference, missing values are iteratively reconstructed through the reverse diffusion process using the learned pseudotime-conditioned denoising model.

**Algorithm 1** Pseudotime-Conditioned CSDI (P-CSDI)

**Require:** Incomplete data $x_{0}$, mask m, diffusion steps *T*

**Ensure:** Imputed sample ${\hat{x}}_{0}^{\text{mis}}$

1: **Pseudotime Estimation:** 

2: Estimate pseudotime τ from $x_{0}$ using trajectory inference methods

3: Reorder data according to τ

4: **Training Procedure:** 

5: **while** not converged **do** 

6:   Sample $x_{0}$, m, and τ

7:   Split $x_{0}$ into $x_{0}^{\text{obs}}$ and $x_{0}^{\text{mis}}$

8:   Sample t∼U({1,…,T}) and ϵ∼N(0,I)

9:   Diffuse missing entries:

$x_{t}^{\text{mis}}=\sqrt{{\bar{\alpha }}_{t}}x_{0}^{\text{mis}}+\sqrt{1-{\bar{\alpha }}_{t}}\epsilon$

10:   Predict noise: $\epsilon_{\theta }(x_{t}^{\text{mis}},t|x_{0}^{\text{obs}},\tau )$

11:   Update θ by minimizing the objective

${\left\|\epsilon -\epsilon_{\theta }(x_{t}^{\text{mis}},t|x_{0}^{\text{obs}},\tau )\right\|}_{2}^{2}$

12: **end while** 

13: **Imputation Procedure:** 

14: Initialize $x_{T}^{\text{mis}}∼N(0,I)$

15: **for**  t=T,…,1  **do** 

16:   Sample ϵ∼N(0,I)

17:   Compute:

$\begin{array}{cc} \mu_{\theta } & =\frac{1}{\sqrt{\alpha_{t}}}\left(x_{t}^{\text{mis}}-\frac{1-\alpha_{t}}{\sqrt{1-{\bar{\alpha }}_{t}}}\epsilon_{\theta }(x_{t}^{\text{mis}},t|x_{0}^{\text{obs}},\tau )\right)\\ \sigma_{t}^{2} & =\frac{1-{\bar{\alpha }}_{t-1}}{1-{\bar{\alpha }}_{t}}(1-\alpha_{t}) \end{array}$

18:  Sample:

$x_{t-1}^{\text{mis}}∼N(\mu_{\theta },\sigma_{t}^{2}I)$

19: **end for** 

20: **return**  ${\hat{x}}_{0}=x_{0}^{\text{obs}}\cup {\hat{x}}_{0}^{\text{mis}}$

### 2.3 PH-CSDI: pseudotime-conditioned hybrid-noise diffusion model for imputation

Most diffusion-based imputation methods (Tashiro *et al*. 2021, Chen *et al*. 2023) adopt isotropic Gaussian white noise, which lacks temporal structure and may fail to preserve frequency-dependent patterns in time-series single-cell data. To overcome this limitation, our prior work (Bishop *et al*. 2025) introduced a time-varying blue noise-based conditional diffusion model (tBN-CSDI), which demonstrated superior performance over existing approaches. Unlike white noise, blue noise concentrates its spectral power at higher frequencies, inducing structured correlations that emphasize local variations while suppressing low-frequency trends.

To capture both continuous cellular dynamics and frequency-dependent structures, we further introduce a PH-CSDI. This framework interpolates between white and blue noise in both the forward and reverse diffusion processes while incorporating pseudotime in the reverse process, enabling trajectory-aware and frequency-aware imputation. Similar to our previous tBN-CSDI framework (Bishop *et al*. 2025), the injected hybrid noise at each diffusion step *t* is defined as a time-varying interpolation between white and blue noise:


(8) $${\tilde{\epsilon }}_{t}=\gamma_{t}\epsilon_{\text{white}}+(1-\gamma_{t})\epsilon_{\text{blue}},$$

where $\epsilon_{\text{blue}}$ denotes structured blue noise generated using the spectral blue-noise construction procedure described in our prior work. Briefly, blue-noise samples are obtained by transforming white Gaussian noise with a precomputed lower-triangular Cholesky factor $L_{\text{blue}}$ (Huang *et al*. 2024), i.e. $\epsilon_{\text{blue}}=L_{\text{blue}}\epsilon_{\;\text{white}}.$ We refer readers to (Bishop *et al*. 2025) for full details of the blue-noise covariance construction and generation procedure.

The blending coefficient $\gamma_{t}\in [0,1]$ depends only on the diffusion step *t*, providing a time-varying interpolation between white and blue noise. Several scheduling strategies can be used to define $\gamma_{t}$, including step, linear, and logistic (sigmoid) schedules. Our recent work observed that model performance is relatively insensitive to the specific choice of noise schedule, although the sigmoid/logistic schedule consistently yielded slightly better results. We continue to evaluate multiple schedule types in the present study. For completeness, the candidate schedules considered are defined as follows:

**Step schedule:**


$$\gamma_{t}=\{\begin{array}{cc} \gamma_{\text{start}}, & t<aT,\\ \gamma_{\text{end}}, & t\geq aT, \end{array}$$

where a∈(0,1) controls the switching point.

**Linear schedule:**


$$\gamma_{t}=\gamma_{\text{start}}+(\gamma_{\text{end}}-\gamma_{\text{start}})\frac{t}{T}.$$

**Logistic/sigmoid schedule:**


$$\gamma_{t}=\sigma \left[\gamma_{\text{start}}+(\gamma_{\text{end}}-\gamma_{\text{start}}){\left(\frac{t}{T}\right)}^{\gamma_{\tau }}\right],$$

where *T* is the total number of steps, and $\gamma_{\text{start}}$, $\gamma_{\text{end}}$, and $\gamma_{\tau }>0$ are hyperparameters controlling the shape of the schedule, and σ(·) is a sigmoid function ensuring $\gamma_{t}\in [0,1]$.

Following P-CSDI and tBN-CSDI, the forward diffusion process is applied only to the missing entries, and the noisy sample at diffusion step *t* is generated using the hybrid noise ${\tilde{\epsilon }}_{t}$ defined in Equation (8), and is expressed as


(9) $$x_{t}^{\text{mis}}=\sqrt{{\bar{\alpha }}_{t}}x_{0}^{\text{mis}}+\sqrt{1-{\bar{\alpha }}_{t}}{\tilde{\epsilon }}_{t}.$$

The reverse diffusion process performs imputation by iteratively updating only the missing components while conditioning on observed values and pseudotime, thereby enabling trajectory-aware denoising under the hybrid noise formulation:


(10) $$x_{t-1}^{\text{mis}}=\frac{1}{\sqrt{\alpha_{t}}}\left(x_{t}^{\text{mis}}-\frac{1-\alpha_{t}}{\sqrt{1-{\bar{\alpha }}_{t}}}{\tilde{\epsilon }}_{\theta }(x_{t}^{\text{mis}},t|x_{0}^{\text{obs}},\tau )\right)+\sigma_{t}{\tilde{\epsilon }}_{t},$$

where ${\tilde{\epsilon }}_{\theta }(\cdot )$ denotes a pseudotime-conditioned neural network that predicts the hybrid noise component given the observed entries and the inferred cellular trajectory coordinate τ.

The PH-CSDI is trained by optimizing the neural network ${\tilde{\epsilon }}_{\theta }$ to minimize the diffusion loss defined as the mean squared error between the true and predicted hybrid noise:


(11) $$\underset{\theta }{\min}E_{x_{0},\tau ,t,\tilde{\epsilon }}\left[{\left\|{\tilde{\epsilon }}_{t}-{\tilde{\epsilon }}_{\theta }(x_{t}^{\text{mis}},t|x_{0}^{\text{obs}},\tau )\right\|}_{2}^{2}\right].$$

Algorithm 2 summarizes the training and imputation procedures of the proposed PH-CSDI. Compared with Algorithm 1, the key difference is that PH-CSDI replaces standard Gaussian white noise with a time-varying hybrid noise that interpolates between white and blue noise across diffusion steps. By integrating pseudotime conditioning with hybrid-noise diffusion, PH-CSDI captures both trajectory-dependent and frequency-dependent structures during denoising.

**Algorithm 2** Pseudotime-conditioned Hybrid-Noise CSDI (PH-CSDI)

**Require:** Incomplete data $x_{0}$, mask m, diffusion steps *T*, hybrid-noise schedule $\gamma_{t}$, precomputed Cholesky factor $L_{\text{blue}}$

**Ensure:** Imputed sample ${\hat{x}}_{0}$

1: **Pseudotime Estimation:** 

2: Estimate pseudotime τ from $x_{0}$

3: Reorder data according to τ

4: **Training Procedure:** 

5: **while** not converged **do** 

6:   Sample minibatch $x_{0},m,\tau$, and t∼U({1,…,T})

7:   Split $x_{0}$ into $x_{0}^{\mathrm{obs}},x_{0}^{\mathrm{mis}}$

8:   Sample white noise $\epsilon_{\mathrm{white}}∼N(0,I)$

9:   Generate blue noise $\epsilon_{\mathrm{blue}}=L_{\mathrm{blue}}\epsilon_{\mathrm{white}}$

10:    Compute hybrid noise ${\tilde{\epsilon }}_{t}=\gamma_{t}\epsilon_{\mathrm{white}}+(1-\gamma_{t})\epsilon_{\mathrm{blue}}$

11:    Diffuse missing entries:

$x_{t}^{\mathrm{mis}}=\sqrt{{\bar{\alpha }}_{t}}x_{0}^{\mathrm{mis}}+\sqrt{1-{\bar{\alpha }}_{t}}{\tilde{\epsilon }}_{t}$

12:   Predict noise: ${\tilde{\epsilon }}_{\theta }(x_{t}^{\mathrm{mis}},t|x_{0}^{\mathrm{obs}},\tau )$

13:   Update θ by minimizing

${\left\|{\tilde{\epsilon }}_{t}-{\tilde{\epsilon }}_{\theta }(x_{t}^{\mathrm{mis}},t|x_{0}^{\mathrm{obs}},\tau )\right\|}_{2}^{2}$

14: **end while** 

15: **Imputation Procedure:** 

16: Initialize $x_{T}^{\mathrm{mis}}∼N(0,I)$

17: **for**  t=T to 1 **do** 

18:   Sample $\epsilon_{\mathrm{white}}∼N(0,I)$

19:   Generate $\epsilon_{\mathrm{blue}}=L_{\mathrm{blue}}\epsilon_{\mathrm{white}}$

20:   Compute hybrid noise ${\tilde{\epsilon }}_{t}=\gamma_{t}\epsilon_{\mathrm{white}}+(1-\gamma_{t})\epsilon_{\mathrm{blue}}$

21:   Compute posterior mean

${\tilde{\mu }}_{\theta }=\frac{1}{\sqrt{\alpha_{t}}}\left(x_{t}^{\mathrm{mis}}-\frac{1-\alpha_{t}}{\sqrt{1-{\bar{\alpha }}_{t}}}{\tilde{\epsilon }}_{\theta }(x_{t}^{\mathrm{mis}},t|x_{0}^{\mathrm{obs}},\tau )\right)$

22:   **if**  t>1  **then** 

23:    Compute $\sigma_{t}$

24:    Sample $x_{t-1}^{\mathrm{mis}}={\tilde{\mu }}_{\theta }+\sigma_{t}{\tilde{\epsilon }}_{t}$

25:   **else** 

26:    Set $x_{0}^{\mathrm{mis}}={\tilde{\mu }}_{\theta }$

27:   **end if** 

28: **end for** 

29: **return**  ${\hat{x}}_{0}=x_{0}^{\mathrm{obs}}\cup x_{0}^{\mathrm{mis}}$

It is worth noting that the proposed methods do not introduce any modifications to the underlying noise prediction network. Specifically, P-CSDI employs the same conditional score network as the original CSDI framework (Tashiro *et al*. 2021), while PH-CSDI adopts the same conditional score network as tBN-CSDI (Bishop *et al*. 2025). In both cases, the denoising network consists of a stack of Transformer-based residual blocks that jointly encode the noisy input, diffusion-step embedding, and conditioning information to predict the additive noise at each reverse diffusion step. Therefore, the performance improvements achieved by P-CSDI and PH-CSDI are attributable to the incorporation of pseudotime conditioning and, for PH-CSDI, the hybrid-noise diffusion mechanism, rather than to changes in the neural network architecture.

Before applying the proposed pseudotime-conditioned diffusion models for imputation (Algorithms 1 and 2), pseudotime is first estimated to provide a continuous representation of cellular progression for downstream conditioning. In the following, we describe the pseudotime estimation procedure and the corresponding cell reordering strategy based on different trajectory inference methods (Haghverdi *et al*. 2016, Street *et al*. 2018, Moon *et al*. 2019).

### 2.4 Pseudotime estimation and cell reordering

Let $X\in R^{n\times G}$ denote the gene expression matrix of scRNA-seq, where *n* is the number of cells and *G* is the number of genes, and let $h_{i}$ denote the observed experimental time label for cell *i*. Because cells collected at the same experimental time may occupy different developmental states, the observed labels may not accurately reflect true biological progression. To obtain a continuous temporal representation of cellular dynamics, we estimate a pseudotime coordinate $\tau_{i}\in [0,1]$ for each cell and reorder cells accordingly for downstream pseudotime-conditioned diffusion modeling.

For all pseudotime methods considered, we first apply a common preprocessing pipeline consisting of gene filtering, highly variable gene selection, standardization, and principal component analysis (PCA) to obtain a low-dimensional representation of the cells. Pseudotime is then inferred from this representation using one of three trajectory inference methods: DPT (Haghverdi *et al*. 2016), Slingshot (Street *et al*. 2018), or Potential of Heat-diffusion for Affinity-based Trajectory Embedding (PHATE) (Moon *et al*. 2019). For DPT, root cells are selected from the subset of cells corresponding to the earliest experimental time point, i.e. $R_{0}=\{i:h_{i}={\text{min}}_{j}h_{j}\}$. To improve robustness to root selection, we randomly sample multiple roots from $R_{0}$, compute a pseudotime trajectory for each root, and aggregate the resulting trajectories by their median to obtain a consensus pseudotime ordering. The final pseudotime values are then normalized to the unit interval. For Slingshot and PHATE, pseudotime is inferred from the same PCA embedding, oriented according to experimental time labels when necessary, and normalized to the unit interval for consistency.

To obtain biologically meaningful temporal ordering for diffusion-based imputation, we first infer pseudotime globally across all cells in the time-series scRNA-seq dataset, assigning each cell a latent trajectory coordinate $\tau_{i}\in [0,1]$. To preserve the original experimental sampling structure, cells are not reordered across time points; instead, within each experimental time bin $h_{t}$, cells are sorted according to their inferred pseudotime values. After reordering, the temporal sample provided to the diffusion model for row index *i* is


$$(x^{h_{1}}(\tau_{i}),x^{h_{2}}(\tau_{i}),\ldots ,x^{h_{T}}(\tau_{i})),$$

where $x^{h_{t}}(\tau_{i})$ denotes the cell in time bin $h_{t}$ whose pseudotime rank approximately matches latent progression level $\tau_{i}$. Consequently, the *i*-th row across time bins forms an approximate pseudo-trajectory of biologically comparable cellular states, yielding smoother temporal transitions and improved temporal coherence in the input sequences.

After pseudotime estimation, cells are reordered according to their inferred developmental progression, and the resulting pseudotime-ordered samples are used as input to the conditional diffusion model. In our framework, pseudotime conditioning is implemented through this pseudotime-guided cell reordering rather than as a separate algorithmic component. This trajectory-aware organization enables the denoising network to learn gene expression patterns that evolve smoothly along the inferred developmental trajectory rather than across heterogeneous cell populations, improving temporal coherence in the input sequences and facilitating the learning of conditional dependencies during diffusion training. Consequently, the model reconstructs missing values in a manner that is more consistent with the underlying biological progression.

**Figure 1:**
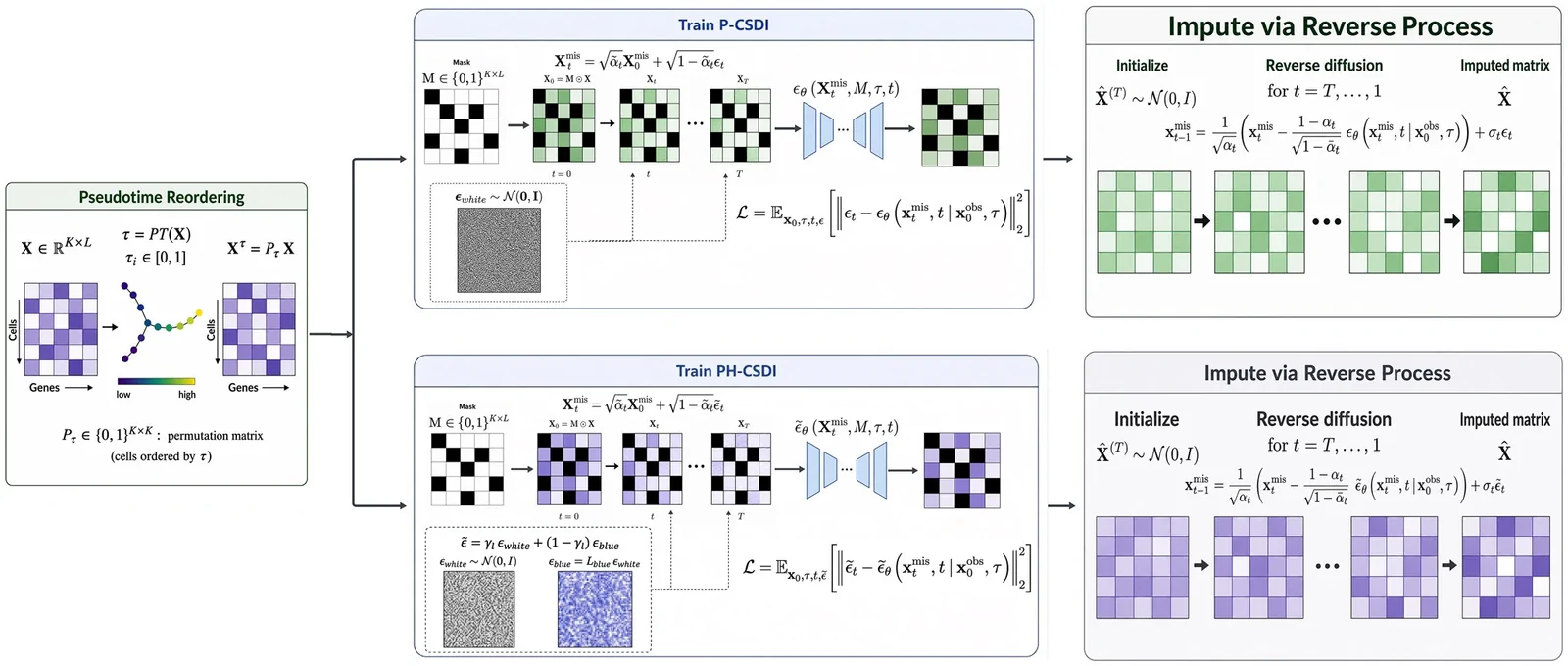
Architecture of the pseudotime-conditioned diffusion models for imputation of missing values in time-series data, including P-CSDI (top) and PH-CSDI (bottom), which encompasses pseudotime estimation and cell reordering, diffusion model training and imputation processes.

Figure 1 presents the overall workflow of the proposed pseudotime-conditioned diffusion frameworks for missing value imputation in time-series data, including P-CSDI (top) and PH-CSDI (bottom). Each framework consists of three main components: pseudotime estimation and cell reordering, diffusion model training, and imputation, corresponding to Algorithms 1 and 2, respectively. The primary distinction between P-CSDI and PH-CSDI is that PH-CSDI additionally incorporates blue-noise generation and hybrid noise interpolation within the forward diffusion (training) and reverse denoising (imputation) processes to better capture frequency-dependent temporal structures.

## 3 Results

In this section, we apply the proposed pseudotime-conditioned diffusion models for imputation to real-world time-series scRNA-seq datasets and evaluate their performance against state-of-the-art methods.

### 3.1 Dataset and evaluation metrics

We evaluate both P-CSDI and PH-CSDI on two widely used real-world time-series scRNA-seq benchmark datasets previously adopted in scRNA-seq analysis studies (Tashiro *et al*. 2021, Bishop *et al*. 2025, Wang *et al*. 2025): (i) THP-1 dataset (Kouno *et al*. 2013) profiles the expression of 45 genes associated with cellular differentiation in 120 monocytic THP-1 human myeloid leukemia cells collected at each of eight time points (0, 1, 6, 12, 24, 48, 72, and 96 h) following stimulation. (ii) mESC dataset (Hayashi *et al*. 2018) profiles mouse embryonic stem cells (mESC) undergoing differentiation into primitive endoderm cells, and contains thousands of genes across 421 single cells sampled at five time points (0, 12, 24, 48, and 72 hr).

To comprehensively evaluate the performance of the proposed methods, we considered both pointwise metrics, including root mean squared error (RMSE) and mean absolute error (MAE), which measure the accuracy of recovered expression values, and the probabilistic metric continuous ranked probability score (CRPS; Tashiro *et al*. 2021), which assesses the quality of uncertainty quantification. Model performance was evaluated using these metrics, consistent with our prior work (Bishop *et al*. 2025) and related studies (Tashiro *et al*. 2021). RMSE and MAE quantify the reconstruction accuracy of temporal gene expression patterns, whereas CRPS measures the discrepancy between the predicted cumulative distribution function and the observed values, providing an assessment of probabilistic calibration. Together, these complementary metrics enable a robust and consistent comparison between the proposed pseudotime-conditioned frameworks and existing time-based models across different missingness levels, rather than relying on individual examples. Lower RMSE, MAE, and CRPS values indicate improved imputation accuracy and more reliable uncertainty estimation.

### 3.2 Experimental setup

Following the experimental protocol of our prior work (Bishop *et al*. 2025), we evaluated the robustness of the proposed pseudotime-conditioned frameworks under varying levels of data sparsity by randomly masking observed values at rates of 10%, 30%, 50%, 70%, and 90%. The data were partitioned into training (60%), validation (20%), and test (20%) sets. The proposed diffusion-based models were trained and evaluated using an NVIDIA T4 GPU on Google Colab, whereas conventional imputation methods were run on a standard laptop.

To assess robustness to pseudotime estimation uncertainty, we further evaluated the proposed framework using pseudotime trajectories inferred by three representative methods: DPT, Slingshot, and PHATE. This analysis examines the sensitivity of imputation performance to different trajectory inference strategies. For each pseudotime method and missingness level, the P-CSDI and PH-CSDI were independently trained and evaluated over five runs. Imputation performance was evaluated using MAE, RMSE, and CRPS, with mean and standard deviation reported across runs. We compared the proposed pseudotime-conditioned methods against several state-of-the-art imputation approaches, including vanilla CSDI, tBN-CSDI (Bishop *et al*. 2025), KNN, SoftImpute (Hastie *et al*. 2015) and MAGIC (Van Dijk *et al*. 2018). Prior work by Tashiro *et al*. (2021) demonstrated that CSDI outperformed SoftImpute, V-RIN, BRITS, and GLIMA (Cao *et al*. 2018, Suo *et al*. 2020) for missing value imputation. Therefore, these methods provide strong and representative baselines for evaluating the proposed framework.

### 3.3 Imputation of THP-1 scRNA-seq data

Table 1 summarizes the imputation performance of the proposed P-CSDI and PH-CSDI methods using DPT-based pseudotime estimation, compared with canonical time-based baseline methods, including tBN-CSDI, CSDI, KNN, SoftImpute and MAGIC, on the THP-1 scRNA-seq dataset. Baseline results for tBN-CSDI, CSDI, KNN, and SoftImpute were adopted from our prior study (Bishop *et al*. 2025), in which tBN-CSDI achieved state-of-the-art performance among existing canonical time-based diffusion and non-diffusion imputation methods. For PH-CSDI, we evaluated two hybrid noise blending schedules: sigmoid and linear. Sigmoid blending consistently yielded slightly better performance across nearly all missingness levels, suggesting a more effective transition between white and blue noise. Therefore, unless otherwise noted, subsequent PH-CSDI results use the sigmoid blending schedule.

**Table 1:** Performance comparison (MAE and RMSE) of the DPT-based pseudotime-conditioned imputation methods P-CSDI and PH-CSDI against baseline approaches, including tBN-CSDI, CSDI, KNN, SoftImpute, and MAGIC, on the THP-1 scRNA-seq dataset across varying missing rates.^a^

Metric	Rate	P-CSDI	PH-CSDI-S	PH-CSDI-L	tBN-CSDI	CSDI	KNN	SoftImpute	MAGIC
MAE	0.1	0.351	0.349	0.365	0.403	0.634	0.867	0.769	0.708
		(0.006)	(0.013)	(0.008)	(0.005)	(0.010)	(0.019)	(0.016)	(0.022)
	0.3	0.359	0.358	0.368	0.391	0.642	0.876	0.775	0.721
		(0.008)	(0.014)	(0.010)	(0.002)	(0.008)	(0.013)	(0.012)	(0.020)
	0.5	0.359	0.364	0.384	0.390	0.641	0.916	0.784	0.746
		(0.004)	(0.006)	(0.007)	(0.005)	(0.007)	(0.006)	(0.008)	(0.018)
	0.7	0.369	0.369	0.394	0.397	0.650	0.989	0.793	0.759
		(0.008)	(0.006)	(0.009)	(0.007)	(0.003)	(0.009)	(0.006)	(0.018)
	0.9	0.381	0.384	0.406	0.412	0.672	0.858	0.792	0.764
		(0.004)	(0.006)	(0.006)	(0.003)	(0.004)	(0.004)	(0.002)	(0.022)
RMSE	0.1	0.500	0.494	0.509	0.578	0.918	1.110	0.995	0.946
		(0.011)	(0.017)	(0.016)	(0.009)	(0.010)	(0.029)	(0.025)	(0.053)
	0.3	0.511	0.507	0.520	0.564	0.932	1.138	1.020	0.956
		(0.009)	(0.019)	(0.012)	(0.008)	(0.005)	(0.018)	(0.015)	(0.039)
	0.5	0.508	0.517	0.542	0.558	0.931	1.197	1.028	0.983
		(0.005)	(0.012)	(0.009)	(0.009)	(0.008)	(0.012)	(0.012)	(0.036)
	0.7	0.524	0.525	0.556	0.563	0.931	1.303	1.038	0.994
		(0.012)	(0.004)	(0.012)	(0.009)	(0.003)	(0.018)	(0.009)	(0.034)
	0.9	0.539	0.541	0.565	0.587	0.962	1.143	1.040	1.000
		(0.006)	(0.008)	(0.010)	(0.006)	(0.003)	(0.012)	(0.005)	(0.037)

a PH-CSDI-S and PH-CSDI-L denote PH-CSDI with sigmoid and linear noise blending schedules, respectively. Results are reported as the mean (standard deviation) over five independent trials.

Our results show that the proposed pseudotime-conditioned diffusion models consistently outperform all baseline methods across every missingness level. Compared with vanilla CSDI, the proposed methods achieve improvements exceeding 40% in most settings. For example, under 10% missingness, PH-CSDI reduces MAE and RMSE by 44.9% and 46.2%, respectively. Relative to the previous best-performing diffusion model, tBN-CSDI, PH-CSDI further reduces MAE from 0.403 to 0.349 (13.4%) and RMSE from 0.578 to 0.494 (14.5%) under 10% missingness, with similarly consistent improvements across higher missingness levels. The gains over conventional imputation methods are even larger. Compared with KNN, the proposed methods reduce MAE and RMSE by up to 59.7% and 55.5%, respectively, while achieving approximately 50% reductions relative to both SoftImpute and MAGIC. These results demonstrate that incorporating pseudotime information into diffusion models substantially improves the recovery of nonlinear developmental dynamics in single-cell transcriptomic data.

Table 2 reports the CRPS of the DPT-based pseudotime-conditioned diffusion models together with those of the canonical diffusion models tBN-CSDI and CSDI on the THP-1 scRNA-seq dataset. The proposed methods consistently outperform vanilla CSDI across all missingness levels, demonstrating that pseudotime conditioning improves not only pointwise reconstruction accuracy but also the calibration and sharpness of predictive imputation distributions. For example, under 10% missingness, PH-CSDI reduces CRPS from 0.623 to 0.400, corresponding to a 35.8% improvement over CSDI. Compared with the stronger tBN-CSDI baseline, P-CSDI and PH-CSDI achieve comparable or slightly better CRPS values across all sparsity levels, with PH-CSDI performing best under low missingness while remaining highly competitive as missingness increases. These results indicate that pseudotime-conditioned diffusion modeling preserves predictive uncertainty while substantially improving deterministic imputation accuracy. Although the CRPS improvements over tBN-CSDI are smaller and less consistent than the improvements observed for MAE and RMSE, this is likely because tBN-CSDI already produces well-calibrated predictive distributions, leaving limited room for further improvement in probabilistic calibration. The proposed pseudotime conditioning and hybrid-noise strategy primarily enhance reconstruction accuracy by incorporating biological progression information while maintaining the uncertainty quantification capability of diffusion-based models.

**Table 2:** Comparison of CRPS, training time (TrainT), and inference time (InferT) for the DPT-based pseudotime-conditioned imputation methods (P-CSDI and PH-CSDI) and the baseline diffusion models (tBN-CSDI and CSDI) on the THP-1 scRNA-seq dataset across varying missing rates.^a^

Rate	P-CSDI	TrainT (P)	InferT (P)	PH-CSDI	TrainT (PH)	InferT (PH)	tBN-CSDI	CSDI	Time (CSDI)
0.1	0.403	53.72	98.40	0.400	54.79	99.34	0.408	0.623	124.0
	(0.016)	(0.16)	(0.39)	(0.011)	(2.34)	(1.13)	(0.002)	(0.006)	(1.53)
0.3	0.395	54.32	98.71	0.400	54.04	99.60	0.394	0.618	122.9
	(0.007)	(0.31)	(0.41)	(0.010)	(0.45)	(0.83)	(0.002)	(0.006)	(1.69)
0.5	0.403	54.18	98.48	0.406	54.91	99.84	0.393	0.620	125.3
	(0.006)	(0.26)	(0.42)	(0.006)	(0.47)	(0.72)	(0.003)	(0.005)	(2.91)
0.7	0.411	54.58	98.42	0.412	54.65	99.60	0.397	0.627	124.6
	(0.008)	(0.54)	(0.53)	(0.007)	(0.35)	(0.76)	(0.004)	(0.003)	(2.13)
0.9	0.427	55.82	99.76	0.428	54.62	98.65	0.401	0.643	124.1
	(0.002)	(0.50)	(1.01)	(0.005)	(0.37)	(0.18)	(0.002)	(0.002)	(0.98)

a The total runtime of CSDI was obtained from our prior work for comparison. Results are reported as the mean (standard deviation) over five independent trials. The runtime is reported in seconds.

Table 2 also reports the runtime of P-CSDI and PH-CSDI, together with the runtime of CSDI reported in our previous work (Bishop *et al*. 2025). Conventional methods (KNN, SoftImpute, and MAGIC) complete within a few seconds, whereas P-CSDI and PH-CSDI require higher computational costs due to the iterative denoising procedures involved in both training and inference. Their runtimes are also slightly higher than those of CSDI and tBN-CSDI (Bishop *et al*. 2025), reflecting the additional computational overhead introduced by the pseudotime-conditioned diffusion framework. Moreover, runtime remains nearly unchanged across different missing rates, indicating that data sparsity has limited impact on computational cost. Since MAE, RMSE, and CRPS are computed simultaneously during evaluation, the reported runtimes are identical across these metrics. Although the proposed methods are computationally more expensive than conventional approaches, they achieve substantially improved imputation accuracy, demonstrating a favorable trade-off between computational cost and predictive performance.

Table 3 evaluates the proposed pseudotime-conditioned diffusion models using two alternative pseudotime inference methods, PHATE and Slingshot, to assess the sensitivity of model performance to the choice of pseudotime estimation algorithm. The imputation results obtained with PHATE- and slingshot-derived pseudotime are highly comparable across all missingness levels and evaluation metrics, indicating that the proposed pseudotime-conditioned framework is robust to the specific pseudotime inference method employed. This suggests that the performance gains of P-CSDI and PH-CSDI arise primarily from leveraging biologically meaningful developmental ordering rather than depending on a particular pseudotime estimation technique. Consistent with the observations in Table 1, the sigmoid-based hybrid noise schedule continues to outperform the linear schedule in nearly all settings, further supporting sigmoid blending as the preferred noise interpolation strategy for PH-CSDI.

**Table 3:** Imputation performance (MAE, RMSE, CRPS) of PHATE-based and Slingshot-based pseudotime-conditioned imputation methods (P-CSDI, PH-CSDI) on THP-1 scRNA-seq data with different missing rates.^a^

Pseudotime	Rate	**P-CSDI**			**PH-CSDI (sigmoid)**			**PH-CSDI (linear)**		
		MAE	RMSE	CRPS	MAE	RMSE	CRPS	MAE	RMSE	CRPS
PHATE	0.1	0.349	0.481	0.380	0.341	0.473	0.378	0.365	0.503	0.398
		(0.007)	(0.013)	(0.008)	(0.008)	(0.015)	(0.011)	(0.008)	(0.008)	(0.015)
	0.3	0.347	0.479	0.374	0.349	0.482	0.376	0.373	0.508	0.396
		(0.007)	(0.010)	(0.006)	(0.007)	(0.010)	(0.004)	(0.010)	(0.015)	(0.007)
	0.5	0.358	0.493	0.382	0.355	0.490	0.382	0.375	0.516	0.397
		(0.006)	(0.007)	(0.006)	(0.004)	(0.002)	(0.007)	(0.004)	(0.007)	(0.003)
	0.7	0.372	0.513	0.395	0.368	0.507	0.392	0.392	0.536	0.410
		(0.004)	(0.006)	(0.003)	(0.010)	(0.014)	(0.005)	(0.002)	(0.006)	(0.001)
	0.9	0.378	0.519	0.400	0.376	0.518	0.398	0.400	0.548	0.415
		(0.005)	(0.004)	(0.004)	(0.004)	(0.006)	(0.002)	(0.005)	(0.006)	(0.004)
Slingshot	0.1	0.357	0.507	0.387	0.350	0.494	0.383	0.364	0.515	0.401
		(0.015)	(0.019)	(0.012)	(0.018)	(0.024)	(0.008)	(0.019)	(0.025)	(0.011)
	0.3	0.358	0.500	0.388	0.348	0.491	0.384	0.369	0.520	0.400
		(0.011)	(0.015)	(0.009)	(0.006)	(0.010)	(0.009)	(0.009)	(0.011)	(0.003)
	0.5	0.360	0.506	0.393	0.360	0.507	0.393	0.384	0.536	0.414
		(0.008)	(0.010)	(0.010)	(0.007)	(0.010)	(0.007)	(0.011)	(0.013)	(0.011)
	0.7	0.381	0.532	0.411	0.375	0.528	0.407	0.395	0.554	0.423
		(0.007)	(0.011)	(0.003)	(0.008)	(0.012)	(0.008)	(0.008)	(0.007)	(0.008)
	0.9	0.407	0.565	0.438	0.402	0.557	0.437	0.420	0.580	0.449
		(0.011)	(0.010)	(0.010)	(0.006)	(0.008)	(0.005)	(0.004)	(0.006)	(0.006)

a Results are reported as mean (std) over five trials.

Overall, the results in Tables 1–3 demonstrate that pseudotime-conditioned diffusion models substantially improve imputation accuracy over both prior state-of-the-art canonical time-based diffusion models and conventional imputation methods. The consistent gains observed across multiple pseudotime inference strategies further indicate that these improvements are robust to the choice of pseudotime estimation algorithm. Collectively, these findings suggest that incorporating biologically inferred developmental progression provides critical structural information for reconstructing missing gene expression values in sparse single-cell RNA-seq (scRNA-seq) data. In contrast to experimental time labels, which may inadequately capture asynchronous cellular dynamics and transcriptomic heterogeneity, pseudotime more accurately reflects the latent developmental continuum underlying the data. Consequently, conditioning diffusion models on pseudotime enables more accurate imputation of missing expression values in dynamic single-cell systems.

### 3.4 Imputation of large-scale mESC scRNA-seq data

The mESC scRNA-seq dataset, which profiles gene expression dynamics during differentiation of mESC into primitive endoderm cells (Hayashi *et al*. 2018), provides a substantially larger and more challenging benchmark than the THP-1 dataset due to its higher dimensionality, greater cellular heterogeneity, and larger proportion of zero-valued entries, offering a rigorous setting for evaluating the generalizability and robustness of the proposed pseudotime-conditioned imputation framework. Consistent with our recent work (Bishop *et al*. 2025), and to mitigate memory limitations of existing imputation methods on high-dimensional data, we restricted the analysis to a representative subset of 100 randomly selected genes.

Table 4 summarizes the MAE and RMSE performance of the DPT-based P-CSDI and PH-CSDI models on the mESC scRNA-seq dataset. Consistent with the THP-1 results, both pseudotime-conditioned methods substantially outperform all baseline approaches across all missingness levels. In most settings, P-CSDI and PH-CSDI reduce MAE and RMSE by over 50% relative to conventional imputation methods such as KNN and SoftImpute. Compared with the prior state-of-the-art tBN-CSDI, PH-CSDI achieves over 30% reductions in both MAE and RMSE across missing rates, with P-CSDI showing similarly strong improvements, further demonstrating the effectiveness of pseudotime conditioning.

**Table 4:** Performance comparison (MAE and RMSE) of DPT-based pseudotime-conditioned imputation methods [P-CSDI and PH-CSDI (Sigmoid)] with baseline approaches, including tBN-CSDI, CSDI, KNN, SoftImpute, and MAGIC on mESC scRNA-seq data across varying missing rates.^a^

Metric	Rate	P-CSDI	PH-CSDI-S	tBN-CSDI	CSDI	KNN	SoftImpute	MAGIC
MAE	0.1	0.304 (0.014)	0.304 (0.012)	0.456 (0.005)	0.727 (0.005)	0.907 (0.020)	0.787 (0.021)	0.491 (0.017)
	0.3	0.305 (0.011)	0.302 (0.004)	0.448 (0.005)	0.730 (0.006)	0.954 (0.019)	0.785 (0.010)	0.576 (0.011)
	0.5	0.306 (0.004)	0.301 (0.006)	0.451 (0.001)	0.725 (0.002)	0.964 (0.015)	0.787 (0.015)	0.671 (0.008)
	0.7	0.309 (0.005)	0.305 (0.005)	0.449 (0.005)	0.727 (0.003)	0.941 (0.018)	0.785 (0.011)	0.783 (0.011)
	0.9	0.314 (0.005)	0.315 (0.004)	0.452 (0.008)	0.732 (0.001)	0.818 (0.015)	0.783 (0.011)	0.851 (0.007)
RMSE	0.1	0.418 (0.024)	0.416 (0.018)	0.590 (0.006)	0.950 (0.005)	1.156 (0.030)	0.974 (0.038)	0.655 (0.022)
	0.3	0.416 (0.013)	0.414 (0.004)	0.587 (0.007)	0.966 (0.008)	1.228 (0.022)	0.993 (0.016)	0.729 (0.021)
	0.5	0.414 (0.007)	0.408 (0.006)	0.592 (0.004)	0.961 (0.002)	1.248 (0.015)	0.993 (0.022)	0.814 (0.012)
	0.7	0.419 (0.006)	0.413 (0.008)	0.590 (0.008)	0.970 (0.003)	1.221 (0.026)	0.992 (0.013)	0.929 (0.013)
	0.9	0.427 (0.007)	0.428 (0.004)	0.594 (0.013)	0.976 (0.004)	1.052 (0.020)	0.995 (0.015)	1.001 (0.008)

a Results are reported as mean (std) over five trials. Lower is better for all metrics.

Table 5 reports CRPS results on the mESC dataset, where the probabilistic advantages of pseudotime conditioning become even more pronounced. Both proposed methods achieve substantially lower CRPS than CSDI and tBN-CSDI across all missingness levels, indicating improved probabilistic calibration and uncertainty quantification. In particular, PH-CSDI reduces CRPS by about 40% compared with tBN-CSDI and by about 60% compared with CSDI. Runtime analysis further shows that both P-CSDI and PH-CSDI are largely insensitive to the missing rate, consistent with the observations on the THP-1 dataset. Despite these strong gains over baseline methods, the performance gap between P-CSDI and PH-CSDI remains small across MAE, RMSE, and CRPS, suggesting that accurate biological trajectory alignment through pseudotime conditioning is the primary driver of performance, whereas additional frequency-dependent noise modeling provides only limited marginal benefit once cells are properly ordered along the pseudotemporal trajectory. Runtime analysis further demonstrates that both methods are insensitive to the degree-of-missingness, as their computational costs remain nearly constant across different missing rates, consistent with the earlier observations.

**Table 5:** Comparison of CRPS, training time (TrainT), and inference time (InferT) for pseudotime-conditioned diffusion models and canonical time-based diffusion models on mESC scRNA-seq data.^a^

Rate	P-CSDI	TrainT (P)	InferT (P)	PH-CSDI	TrainT (PH)	InferT (PH)	tBN-CSDI	CSDI	Time (CSDI)
0.1	0.278	67.40	134.49	0.279	67.87	135.64	0.443	0.695	107.9
	(0.013)	(1.39)	(0.61)	(0.013)	(1.93)	(0.82)	(0.005)	(0.006)	(0.46)
0.3	0.276	67.07	135.00	0.274	67.37	135.89	0.438	0.698	110.0
	(0.008)	(0.16)	(0.37)	(0.004)	(0.38)	(0.80)	(0.002)	(0.004)	(0.44)
0.5	0.276	67.16	135.03	0.273	67.39	135.80	0.444	0.703	110.3
	(0.003)	(0.20)	(0.28)	(0.004)	(0.37)	(0.50)	(0.001)	(0.002)	(0.94)
0.7	0.277	67.40	134.97	0.275	67.07	135.47	0.445	0.708	110.5
	(0.003)	(0.20)	(0.52)	(0.004)	(0.37)	(0.55)	(0.002)	(0.003)	(1.76)
0.9	0.282	67.10	134.74	0.281	67.22	135.76	0.446	0.710	111.7
	(0.003)	(0.23)	(0.30)	(0.002)	(0.50)	(0.68)	(0.003)	(0.001)	(1.39)

a The total runtime of CSDI was obtained from our prior work for comparison. Results are reported as mean (std) over five trials. Lower is better for CRPS. The runtime is reported in seconds.

Table 6 continues to assess the sensitivity of the proposed pseudotime-conditioned diffusion models to different pseudotime inference strategies on the large-scale mESC dataset. Similar to the THP-1 results, PHATE- and Slingshot-based pseudotimes yield highly consistent performance across all metrics and missing rates, with small differences in MAE, RMSE, and CRPS for both P-CSDI and PH-CSDI variants. This agreement on a larger and more challenging dataset further supports that the proposed frameworks do not depend strongly on the specific pseudotime construction method. Instead, performance is primarily governed by the existence of a coherent developmental ordering rather than how it is estimated. Across both settings, PH-CSDI with sigmoid scheduling remains slightly but consistently superior to the linear variant, reinforcing its stability as the preferred configuration in large-scale applications.

**Table 6:** Imputation performance (MAE, RMSE, CRPS) of PHATE-based and Slingshot-based pseudotime-conditioned imputation methods (P-CSDI, PH-CSDI with sigmoid and linear noise schedules) on mESC scRNA-seq data with different missing rates.^a^

Pseudotime	Rate	**P-CSDI**			**PH-CSDI (Sigmoid)**			**PH-CSDI (Linear)**		
		MAE	RMSE	CRPS	MAE	RMSE	CRPS	MAE	RMSE	CRPS
PHATE	0.1	0.310	0.424	0.280	0.303	0.418	0.277	0.318	0.437	0.287
		(0.011)	(0.025)	(0.006)	(0.012)	(0.021)	(0.005)	(0.018)	(0.031)	(0.007)
	0.3	0.317	0.436	0.283	0.312	0.430	0.279	0.325	0.450	0.289
		(0.009)	(0.015)	(0.005)	(0.007)	(0.011)	(0.005)	(0.006)	(0.011)	(0.005)
	0.5	0.316	0.436	0.282	0.310	0.429	0.278	0.335	0.462	0.296
		(0.006)	(0.010)	(0.003)	(0.008)	(0.013)	(0.006)	(0.008)	(0.010)	(0.005)
	0.7	0.324	0.443	0.287	0.321	0.442	0.285	0.335	0.458	0.295
		(0.004)	(0.004)	(0.004)	(0.005)	(0.004)	(0.005)	(0.003)	(0.003)	(0.003)
	0.9	0.328	0.448	0.290	0.327	0.450	0.289	0.349	0.477	0.306
		(0.005)	(0.006)	(0.003)	(0.008)	(0.008)	(0.005)	(0.009)	(0.011)	(0.006)
Slingshot	0.1	0.324	0.443	0.285	0.329	0.447	0.287	0.337	0.459	0.295
		(0.006)	(0.011)	(0.005)	(0.008)	(0.015)	(0.009)	(0.013)	(0.021)	(0.006)
	0.3	0.328	0.450	0.289	0.325	0.444	0.289	0.336	0.461	0.298
		(0.007)	(0.010)	(0.006)	(0.006)	(0.008)	(0.002)	(0.008)	(0.009)	(0.004)
	0.5	0.324	0.445	0.288	0.322	0.442	0.286	0.338	0.464	0.297
		(0.006)	(0.008)	(0.005)	(0.004)	(0.005)	(0.002)	(0.008)	(0.008)	(0.004)
	0.7	0.327	0.449	0.289	0.325	0.444	0.288	0.345	0.470	0.303
		(0.004)	(0.006)	(0.004)	(0.004)	(0.006)	(0.002)	(0.003)	(0.005)	(0.002)
	0.9	0.339	0.465	0.298	0.334	0.458	0.296	0.356	0.487	0.311
		(0.004)	(0.007)	(0.002)	(0.003)	(0.006)	(0.002)	(0.008)	(0.010)	(0.006)

a Results are reported as mean (std) over five trials.

Overall, Tables 4–6 demonstrate that the proposed pseudotime-conditioned diffusion models generalize effectively to the larger and more challenging mESC dataset. Across MAE, RMSE, and CRPS, both P-CSDI and PH-CSDI consistently outperform classical imputation methods and diffusion baselines, with error reductions often exceeding 50% over traditional approaches and substantial gains over tBN-CSDI in both accuracy and uncertainty quantification. The small performance gap between P-CSDI and PH-CSDI suggests that pseudotime alignment is the primary contributor to improvement, while additional noise-structure modeling provides limited marginal benefit. Performance remains stable across DPT, PHATE, and Slingshot pseudotime estimates, indicating robustness to the choice of trajectory inference method.

## 4 Discussion

This work introduces pseudotime-conditioned diffusion models for time-series scRNA-seq imputation and demonstrates that incorporating biologically informed temporal structure substantially improves both point estimation and uncertainty quantification. Across multiple datasets (THP-1 and mESC) and pseudotime inference strategies (DPT, PHATE, and Slingshot), the proposed P-CSDI and PH-CSDI consistently outperform diffusion baselines (CSDI and tBN-CSDI) and classical imputation methods such as KNN and SoftImpute by approximately 40%–60%. Prior work has shown that vanilla CSDI outperforms several established imputation approaches, including V-RIN, BRITS, and GLIMA (Cao *et al*. 2018, Suo *et al*. 2020, Tashiro *et al*. 2021); therefore, the superior performance of P-CSDI and PH-CSDI over CSDI suggests that the proposed methods are also likely to outperform these additional baselines. Performance gains are particularly pronounced on the large-scale mESC dataset, where sparsity and high dimensionality pose greater reconstruction challenges. These improvements in MAE, RMSE, and CRPS demonstrate that aligning the diffusion process with pseudotime provides a more informative inductive bias than conditioning on experimental sampling time alone.

A key finding of this study is the consistent improvement of both proposed methods over tBN-CSDI, previously one of the strongest time-conditioned diffusion baselines. This result suggests that pseudotime provides a more biologically meaningful conditioning signal than chronological or experimental time, particularly in asynchronous cellular systems where cells collected at the same experimental time point may occupy different developmental states. The relatively small performance difference between P-CSDI and PH-CSDI further indicates that accurate trajectory guidance is the primary driver of performance improvement, whereas the specific choice of diffusion noise formulation plays a secondary role. Although hybrid spectral noise can provide additional benefits when trajectory information is unavailable, its relative contribution may diminish once biologically informed pseudotime effectively guides the denoising process. Among the proposed variants, PH-CSDI with sigmoid noise scheduling demonstrates slightly more stable performance, suggesting that smoother noise transitions may better accommodate gradual changes during cellular differentiation. Future studies will further investigate the robustness of pseudotime-conditioned diffusion models under inaccurate or perturbed trajectory estimation to better understand the relative contributions of trajectory guidance and noise design.

Another important observation is the robustness of the proposed framework across different pseudotime inference methods. Results obtained using DPT, PHATE, and Slingshot are highly consistent, indicating that the diffusion model does not critically depend on a specific trajectory inference algorithm. This robustness is particularly valuable in practice because pseudotime estimation is inherently uncertain and different algorithms often produce multiple plausible trajectory reconstructions. The consistent performance across methods suggests that the proposed framework primarily benefits from the existence of a coherent latent developmental ordering rather than the precise choice of pseudotime estimator.

Despite these promising results, the current study has several limitations. First, the proposed framework assumes that cells follow a single underlying differentiation trajectory, where a single pseudotime ordering is inferred and incorporated into the diffusion model. Many biological systems, however, exhibit branching or multiple differentiation lineages. Extending the framework to such settings represents an important direction for future research. One possible strategy is to first identify individual lineages using a trajectory inference method and then perform pseudotime-guided imputation independently within each lineage. Alternatively, trajectory identity could be incorporated as an additional conditioning variable, allowing the diffusion model to jointly learn multiple developmental paths. Imputation accuracy may vary across trajectories depending on branching complexity, lineage-specific sample size, and the quality of trajectory inference, making robust modeling of complex branching differentiation processes an important area for future investigation.

Second, although the proposed framework is not intrinsically restricted by dataset size, applying diffusion-based imputation to large-scale single-cell atlases remains computationally challenging due to the iterative nature of diffusion model training and reverse sampling. Future work will focus on developing scalable optimization strategies and efficient sampling algorithms to enable the application of pseudotime-conditioned diffusion models to increasingly large single-cell datasets.

The third limitation of the proposed framework is its reliance on a two-stage pipeline in which pseudotime is estimated prior to diffusion-based imputation, making the overall performance dependent on the quality of the inferred trajectories. Future work will investigate end-to-end architectures that jointly optimize latent trajectory learning and conditional diffusion, potentially reducing error propagation while enabling more adaptive and biologically consistent representations.

Another promising direction is to integrate change-point detection into pseudotime-guided diffusion models to better capture abrupt transitions in biological processes such as cell cycle progression and immune responses (Groer *et al*. 2015). Current diffusion models generally assume smooth progression along pseudotime and employ a globally shared diffusion schedule. In contrast, many biological systems exhibit discrete shifts in gene expression programs. Incorporating change-point detection methods (Dette *et al*. 2019, Si *et al*. 2024) could partition pseudotime into locally homogeneous segments and enable adaptive diffusion schedules or denoising strategies tailored to each developmental stage. Such an approach could improve the modeling of both continuous developmental progression and abrupt cellular state transitions.

Finally, integrating complementary multi-omics information, such as spatial transcriptomics (Heitz *et al*. 2025, Smelik *et al*. 2025), chromatin accessibility, or epigenetic measurements, may further improve pseudotime estimation and provide richer biological context for diffusion-based imputation. Together, these extensions highlight pseudotime-conditioned diffusion models as a flexible and extensible framework for accurate missing value imputation and probabilistic modeling of dynamic single-cell systems.

## Data Availability

The computer code and data are available on GitHub: https://github.com/gbishop345/pseudotime-CSDI.
